# R2 and R2/R1 hybrid non-autonomous retrotransposons derived by internal deletions of full-length elements

**DOI:** 10.1186/1759-8753-3-10

**Published:** 2012-05-23

**Authors:** Danna G Eickbush, Thomas H Eickbush

**Affiliations:** 1 Department of Biology, University of Rochester, Rochester, NY, 14627, USA

**Keywords:** LINE, Non-autonomous elements, Retrotransposon, Ribozyme, SINE, Template jump

## Abstract

**Background:**

R2 is a non-long terminal repeat (non-LTR) retrotransposable element that inserts site specifically into the 28S genes of the ribosomal (r)RNA gene loci. Encoded at the 5' end is a ribozyme that generates the precise 5' end by self-cleavage of a 28S gene cotranscript. Sequences at the 3' end are necessary for the R2 protein to bind RNA and initiate the target primed reverse transcription (TPRT) reaction. These minimal RNA requirements suggested that if recombination/DNA repair conjoined the 5' and 3' ends of R2, the result would be a non-autonomous element that could survive as long as autonomous R2 elements supplied the TPRT activity.

**Results:**

A PCR-based survey of 39 *Drosophila* species aided by genomic sequences from 12 of these species revealed two types of non-autonomous elements. We call these elements SIDEs (for ‘Short Internally Deleted Elements’). The first consisted of a 5' ribozyme and a 3' end of an R2 element as predicted. Variation at the 5' junctions of the R2 SIDE copies was typical for R2 insertions suggesting their propagation by TPRT. The second class of SIDE contained sequences from R1 elements, another non-LTR retrotransposon that inserts into rRNA gene loci. These insertions had an R2 ribozyme immediately upstream of R1 3' end sequences. These hybrid SIDEs were inserted at the R1 site with 14 bp target site duplications typical of R1 insertions suggesting they used the R1 machinery for retrotransposition. Finally, the survey revealed examples of U12 small nuclear (sn)RNA and tRNA sequences at the 5' end of R2 elements suggesting the R2 reverse transcriptase can template jump from the R2 transcript to a second RNA during TPRT.

**Conclusions:**

The R2 SIDE and R2/R1 hybrid SIDEs are rare examples of non-autonomous retrotransposons in the *Drosophila* genome. Associated non-autonomous elements and *in vivo* template jumps are two additional characteristics R2 shares with other non-LTR retrotransposons such as mammalian L1s. Analysis of the hybrid SIDEs provides supporting evidence that R1 elements, like R2 elements, recognize their 3' untranslated region (UTR) sequences and, thus, belong to the stringent class of non-LTR elements.

## Background

The genomes of all eukaryotes contain examples of transposable elements, sequences that generally appear to be genomic parasites although such sequences are occasionally co-opted for the host's benefit [1,2]. These mobile elements fall into families that differ in basic structure and method of transposition [3,4]. Non-long terminal repeat (non-LTR) retrotransposable elements comprise one of the two major families of mobile elements whose movement requires reverse transcriptase. Their mechanism of integration is different from retrotransposable elements with long terminal repeats in that they use the 3' hydroxyl group at a DNA break to prime reverse transcription of their RNA transcripts; a process termed target primed reverse transcription (TPRT) [5]. Full-length non-LTR elements encode the critical enzymes necessary for generating additional copies in the genome and are, therefore, autonomous. A common occurrence with non-LTR elements is that their insertion machinery is hijacked. The elements that parasitize the retrotransposition machinery of autonomous LINEs (for ‘Long INterspersed Elements’) have been called SINEs (for ‘Short INterspersed Elements’). They are represented by Alu elements in primates although dozens of SINE families have been found in other eukaryotic genomes [6-8]. Several SINEs were in part derived from 7SL RNA; however, with the additional exception of a SINE derived from 5 S ribosomal RNA in zebrafish [9], the majority of SINEs in eukaryotic genomes are derived from tRNA genes [6,10]. While their structure is variable, the characteristic attribute of SINEs is that they are transcribed by RNA polymerase III. Recognition of the SINE transcripts by LINE proteins is necessary for their reverse transcription and insertion into a new site. This is accomplished either by sequence identity at the 3' end between the LINE and its associated SINE (stringent elements) or a less strict recognition of a simple sequence, frequently a poly(A) tail, (relaxed elements) [11-14].

**Figure 1:**
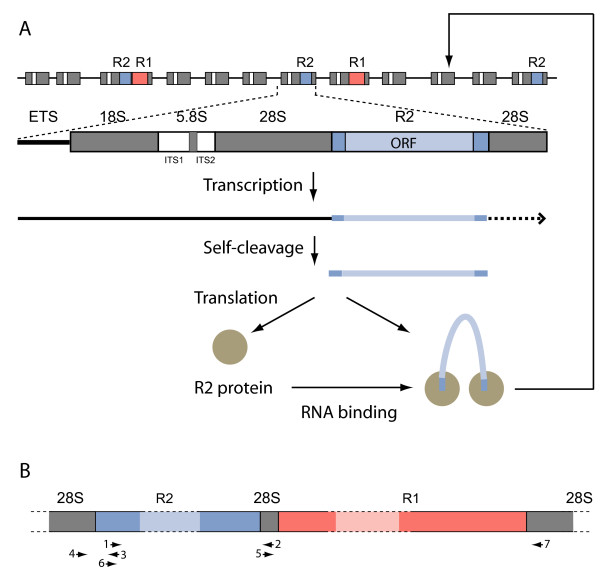
**The rDNA locus and its R2 and R1 element insertions. (A)** The rDNA locus is composed of a tandem array of rDNA units with a subset of these units inserted by R2 (blue boxes) and/or R1 elements (orange boxes). The rRNA transcription unit with external transcribed spacer (ETS), 18S, 5.8S and 28S genes (gray boxes), transcribed spacers (white boxes), and R2 insertion is diagrammed. The single open reading frame (ORF) of R2 is delineated in light blue. R2 RNA sequences are processed from the cotranscript at the 5' end by an R2 encoded self-cleaving ribozyme. After translation, identical subunits of the R2 protein (circles) bind sequences at either end of the R2 transcript, and the RNA/protein complex binds at the R2 target site in the 28S gene and proceeds with the insertion of a new R2 copy. **(B)** Diagram of a portion of the 28S gene with both R2 and R1 insertions. Arrows indicate location and direction of primers in the 28S gene and R2 element used to survey for unusual insertions near the R2 target site.

R2 and R1 are non-LTR retrotransposable elements that insert into specific sites in the 28S ribosomal RNA genes of most animal lineages (Figure 1A) [15]. The mechanism by which non-LTR elements retrotranspose has been best characterized for R2 using the protein encoded by the element in the silk moth, *Bombyx mori*. The R2 transcript has sequences in the 5' untranslated region (UTR) and 3' UTR, which are recognized by the R2 protein although only the sequences in the latter are necessary for insertion of a new copy (Figure 1A). The new copy of the R2 element is inserted into a ribosomal DNA (rDNA) unit via a symmetric series of cleavages of the two DNA strands and utilization of the free ends to prime synthesis [16]. A study of the variation at the junctions of R1 elements suggested that like R2 it is integrated in a series of cleavage and TPRT reactions [17-19]. Both R2 and R1 elements have been extensively studied in *Drosophila* and found to be maintained by vertical descent since the genus arose [20,21]. Analysis of the sequenced genomes of 12 *Drosophila* species indicates that the high sequence identity found among R2 and R1 elements within a species is because all insertions are relatively new [22]. That is, the recombinational forces responsible for the concerted evolution of the rRNA genes rapidly eliminate element copies from the rDNA locus.

While there is no direct evidence, the presence of R1 lineages outside the 28S gene (for example, telomeres) suggests R1 encodes its own promoter [23,24]. R2 elements, however, depend on an encoded self-cleaving ribozyme at their 5' end to process the R2 transcript from a 28S cotranscript. The R2 ribozyme shows remarkable similarity to the structure of the hepatitis delta virus (HDV) ribozyme with many of the conserved nucleotides in *Drosophila* R2 ribozymes identical to residues in the catalytic region of the HDV ribozyme [25].

In our survey of the R2 ribozyme in different species of *Drosophila*, insertions bearing the R2 ribozyme were discovered that did not correspond to the R2 elements of that species. Here we report the discovery of non-autonomous elements with sequence identity to R2 elements as well as multiple examples of hybrid non-autonomous elements with sequence identity to both R2 and R1 elements. Because these elements are not transcribed by polymerase III and therefore not SINEs [6-8], they are referred to as SIDEs (for ‘Short Internally Deleted Elements’). Based on the divergence of their sequence and their abundance, these SIDEs appear active and have persisted for millions of years. Finally, we report evidence for template jumps *in vivo* to small, stable RNAs in the cell, which in one case may have established a new R2 subfamily.

## Results

### R2 SIDE

**Figure 2:**
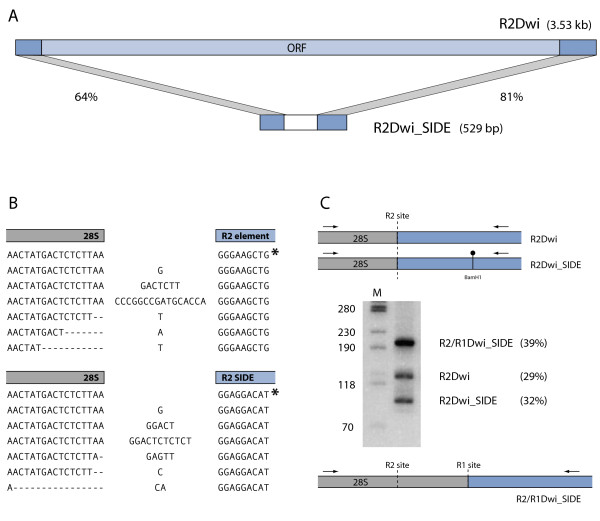
**R2 SIDE (‘Short Internally Deleted Element’) in*Drosophila willistoni.*(A)** The 3.53 kb R2 element in *D. willistoni*, R2Dwi, is diagrammed with the 5' and 3' UTRs (untranslated regions) shaded darker. The 529 bp element, R2Dwi_SIDE, has sequence identity at the 5' and 3' ends to the R2 element (percent identity shown); the 197 bp central region (white box) has no significant identity to the R2 element. **(B)** Sequence reads for full-length R2 and R2Dwi_SIDE elements obtained from the trace archive at NCBI [26]. The majority of 5' junctions for both element types are precise (marked with asterisks). Typical variation at the 5' junction for both elements is also presented. **(C)** Genomic DNA from *D. willistoni* was PCR amplified using a 28S primer (32 nucleotides upstream of the R2 site) and a ribozyme primer (conserved region 100 nucleotides into the elements) (arrows). PCR products after *Bam*HI digestion were separated on a native, 8% polyacrylamide gel. Lane M, DNA length markers with sizes indicated. The product at 200 bp was subsequently determined to correspond to an insertion in the R1 site, R2/R1Dwi_SIDE (bottom diagram). Element type and relative percentage in the genome are to the right of the gel.

While analyzing R2 ribozyme sequences from *Drosophila willistoni*, a sequence located in the R2 insertion site was identified which showed only 64% sequence identity to the 5' UTR of the R2 elements in this species [22]. PCR amplification using a degenerate primer to conserved sequences in the ribozyme paired with a reverse primer to 28S sequences 30 to 50 bp downstream of the R2 site (Figure 1B, primers 1 and 2) generated the expected 3.5 kb R2 element product as well as a much shorter product. Sequencing revealed the short insert had identity to both the 5' and 3' UTRs of the *D. willistoni* R2 and, like R2Dwi, ended in a poly(A) tail. We refer to this insert as a Short Internally Deleted Element, or a SIDE. This particular SIDE is R2Dwi_SIDE to indicate its presence in *D. willistoni* and it relationship to R2. A comparison of the structure of the 3.53 kb *D. willistoni* R2 element to that of the 529 bp R2 SIDE is presented in Figure 2A. Sequence identity at the 5' and 3' ends was 64% and 81% respectively. The central 197 bp of R2Dwi_SIDE showed no apparent identity to R2 or any other *D. willistoni* sequence.

*D. willistoni* was one of the species chosen for the 12 *Drosophila* genomes project [27], thus sequencing reads containing copies of the R2Dwi_SIDE could be obtained from the trace archive. Approximately 70 original reads corresponding to the R2 SIDE were analyzed and found to have minor 5' junction variation and less than 3% nucleotide divergence. As previously documented for R2 element junctions in many *Drosophila* species, most full-length R2 elements in *D. willistoni* insert precisely into the 28S gene. This canonical 5' junction sequence is indicated by an asterisk in the upper portion of Figure 2B. However, many *D. willistoni* R2 element 5' junctions have deletions of the upstream 28S sequences and/or non-templated nucleotide additions. Typical examples of the range of variation are presented below the canonical junction. The full-length R2 SIDE insertions were also found to have a precise, canonical junction and the same range of sequence variation found for the R2 elements. This variation in the 5' junctions as well as variation in the length of the poly (A) tail at the 3' end (13 to 41 A’s for R2; 14 to 38 A’s for the SIDE) suggest that the R2 SIDE in *D. willistoni* is actively using the retrotransposition machinery provided by the autonomous R2 element.

**Figure 3:**
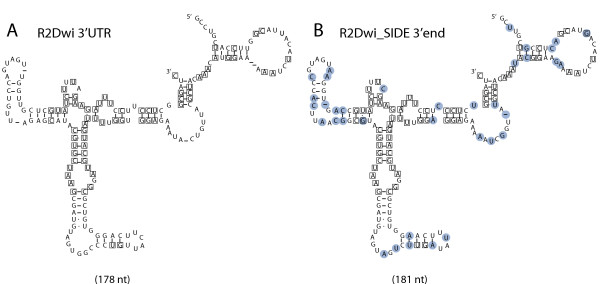
**Secondary structure conservation of R2 3' ends. (A)** RNA sequence from the 3' UTR of the R2 element from *Drosophila willistoni* folded into the secondary structure modeled for other *Drosophila* R2 [28]. Nucleotides identical to those found to be conserved in the previous report are boxed. **(B)** The 3' end sequence from R2Dwi_SIDE folded into the same secondary structure. Nucleotide differences relative to R2Dwi are circled in blue. Boxed nucleotides are as in (A).

This model predicts that the 3' end of the R2 SIDE transcript is recognized by the R2 protein for retrotransposition into a 28S gene (Figure 1A). The secondary structure formed by the 3' UTR RNA of *Drosophila* R2 elements was previously predicted using sequences from ten species in the melanogaster and obscura groups [28]. In Figure 3, it is apparent that both the 3' end of the R2 element and of the R2 SIDE from *D. willistoni* can be folded into this predicted secondary structure. Although these sequences are 20% divergent, nucleotide differences (circled) are largely relegated to the loops or exhibit compensatory changes in base-paired regions. Furthermore, over 90% of the invariant nucleotides found in the previous study are conserved in both element types in *D. willistoni* (boxed nucleotides).

To determine the relative abundance of R2 and R2 SIDE, a PCR primer with sequence identity to both *D. willistoni* elements was used in conjunction with an upstream 28S primer. The R2 element and R2 SIDE products could be differentiated after PCR amplification because the R2 SIDE sequences contain a *Bam*HI restriction site. The PCR results are shown in Figure 2C. The similar intensities of the 130 bp R2 element product and the 97 bp R2 SIDE product after *Bam*HI digestion indicated that they are present in the *D. willistoni* rDNA loci in equal numbers. The unexpected 200 bp PCR product suggested an abundant third element type bearing the R2 ribozyme was also present in the 28S gene at or near the R2 site. The trace archive was searched for the origin of this product. Surprisingly, an element was found with sequence identity to both the R2 ribozyme and the 3' end of the R1 element, forming what appeared to be an R2/R1 hybrid SIDE (R2/R1Dwi_SIDE). A discussion of the R1 component of this hybrid SIDE, which is more abundant than either the full-length R2 or R2Dwi_SIDE, is presented below.

**Figure 4:**
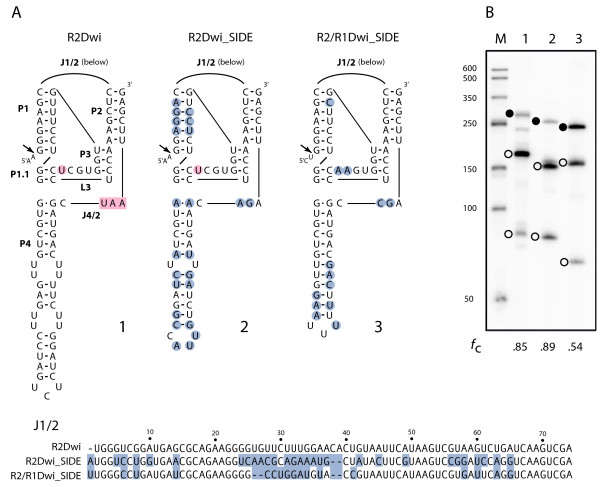
**The 5' ends of*D. willistoni*(Dwi) elements function as ribozymes. (A)** RNA sequences from the 5' end of R2Dwi folded into the secondary structure previously determined for the ribozymes encoded by other *Drosophila* R2 (left). J = nucleotides joining paired regions; L = loop; P = base paired region [25]. Similar structures are presented for R2Dwi_SIDE (‘Short Internally Deleted Element’) (center) and R2/R1Dwi_SIDE (right) with nucleotide differences relative to the R2 element circled in blue. J1/2 sequences for each element type are presented below with nucleotide differences relative to R2Dwi boxed in blue. Nucleotides boxed in pink correspond to a stop codon found in most *Drosophila* R2 elements. Nucleotide circled in pink corresponds to a ‘U’ residue conserved in *Drosophila* R2 elements and the R2 SIDE but not the hybrid SIDEs. **(B)** A 5% polyacrylamide denaturing gel showing the *in vitro* generated RNAs from 5' junction templates starting 95 bp upstream of the R2 site (lanes 1 and 2) or 74 bp upstream of the R1 site (lane 3) and extending 5 to 10 bp downstream of the ribozyme structure. Lane numbers correspond to ribozyme structure in (A). The uncleaved RNA (solid circle) and self-cleaved products (open circles) are indicated for each ribozyme. The fraction of synthesized RNA undergoing self-cleavage (*f*_c_) is under each lane. Lane M, RNA length markers with sizes indicated.

Although 30% to 40% divergent in nucleotide sequence, the secondary structures at the 5' end of R2Dwi_SIDE as well as R2/R1Dwi_SIDE were nearly identical to the R2Dwi ribozyme (Figure 4A). Nucleotide differences, relative to the R2Dwi ribozyme, were predominantly compensatory changes in the four major base-paired regions (P1 to P4) or present in the large J1/2 loop between P1 and P2. Sequences in the J1/2 loop were previously shown to have little effect on self-cleavage of HDV-like ribozymes [25,29]. Each of the three ribozymes was tested in our standard T7 *in vitro* transcription-cleavage assay [25] and each was observed to self-cleave (Figure 4B). The R2 SIDE and the R2 element ribozymes were found to self-cleave at similar levels (89% and 85% respectively), the R2/R1 SIDE ribozyme at a lower level (54%). The lower level of cleavage by the R2/R1 SIDE may be due to the two nucleotide differences in the catalytic L3 region of the ribozyme or the different 28S sequences upstream of the ribozyme. Both types of changes have been shown to affect the level of self-cleavage by HDV-like ribozymes [25,30,31]. The ability to self-cleave suggests that the 5' end of both R2Dwi_SIDE and R2/R1Dwi_SIDE can be processed out of a 28S cotranscript much like the R2 element.

### Survey for additional SIDEs

Several PCR-based surveys were performed to look for additional SIDEs containing the R2 ribozyme in other *Drosophila* species. First, primers 1 and 2 (Figure 1B) gave rise in most of the 39 *Drosophila* species analyzed to a PCR product greater than 3 kb in length consistent with the presence of full-length R2 elements; however, no additional R2 SIDEs were detected. Second, a reverse primer to the catalytic region of the ribozyme was used in conjunction with a primer to 28S sequences upstream of the R2 site to look for PCR products distinct from the full-length R2 product (Figure 1B, primers 3 and 4). This survey also did not reveal additional R2 SIDEs but did lead to the discovery of several examples of *in vivo* template jumps to small cellular RNAs (discussed below). These results suggest R2 SIDEs are not common in *Drosophila*.

A third survey was performed to look for additional hybrid SIDEs in the R1 site of *Drosophila*. Primer 3 was paired with a 28S primer corresponding to sequences between the R2 and R1 sites (Figure 1B, primer 5). This primer pair will only amplify R2 sequences inserted downstream of the R2 site (for example, the R1 site) [22]. PCR products containing R2 sequences were obtained from 11 species. Sequence analysis of the products from eight species suggested that they arose from R2 insertions containing target site duplications greater than 20 bp in length, therefore, only appeared inserted downstream of the R2 site. Such target site duplications have been previously detected for R2 elements [22]. However, an analysis of the products from *Drosophila falleniDrosophila innubila* and *Drosophila immigrans* did reveal additional SIDE elements. The 3' end of each of these insertions was obtained using a species-specific primer paired with a primer downstream of the R1 site (Figure 1B, primer 6 and primer 7).

### R2/R1 SIDEs

**Figure 5:**
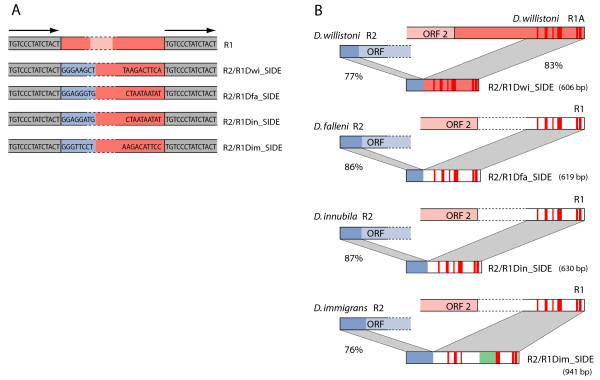
**R2/R1 hybrid SIDEs. (A)** R1 insertions in the 28S gene in *Drosophila* outside the melanogaster group are flanked by a 14 bp target site duplication (arrows, upper diagram). In four species (bottom diagrams), a family of insertion elements bearing R2 ribozyme sequences (blue box) upstream of sequences with identity to R1 elements (orange box) was found in the R1 site flanked by the same 14 bp target site duplication. **(B)** The diagrams show the extent and level of sequence identity of each hybrid SIDE to the R1 and R2 elements in the same species. In the case of the R2/R1 SIDEs from *Drosophila falleni*, *Drosophila innubila* and *Drosophila immigrans* sequence identity to R1 was limited to six conserved segments found in all *Drosophila* R1 elements (red vertical lines; see Additional file 1). A portion of the sequence between the third and fourth conserved segments in R2/R1Dim_SIDE has 75% identity to ITS-1 of *D. immigrans* (green box). The lengths of the R2/R1 SIDEs are shown to the right.

Based on their 3' junctions, all R1 elements within the 28S gene are located 60 bp downstream of the R2 insertion site. Based on their 5' junctions, all R1 elements outside the melanogaster species group have a 14 bp target site duplication that flanks the R1 insertions [22]. The hybrid insertion elements found in *D. willistoni, D. falleniD. innubila* and *D. immigrans* were present in the R1 site and also had a 14 bp target site duplication (Figure 5A). Schematic diagrams of the insertions- R2/R1Dfa_SIDE, R2/R1Din_SIDE, R2/R1Dim_SIDE and R2/R1Dwi_SIDE- are presented in Figure 5B. Sequence identity to R2 for the four hybrid SIDEs was confined to the ribozyme plus five to eight downstream nucleotides and varied from 76% to 87%. Sequence identity to R1 for the 3' ends of the hybrid SIDEs varied from only short segments to 83% in the case of *D. willistoni*. Previous analysis of *Drosophila* R1s has revealed the 3' UTR varies considerably in length between species (500 to 1,000 bp) with little sequence conservation [21]. A detailed comparison of the 3' UTRs of divergent *Drosophila* R1s (Additional file 1) revealed six conserved regions. The R2/R1 SIDEs in *D. willistoniD. falleni*, and *D. innubila* have these six conserved segments spaced at intervals consistent with those observed for R1 elements (Additional file 1; Figure 5B, red vertical bars). Only the hybrid SIDE from *D. immigrans* differed by the addition of extra sequences between the third and fourth conserved segments. Surprisingly, half of this extra sequence appears to be derived from the internal transcribed spacer (ITS)-1 region of the *D. immigrans* rDNA unit (green shading). The conservation of the critical segments at the 3' ends of the R2/R1 SIDEs as well as their target site specificity suggest the hybrid SIDEs use the R1 retrotransposition machinery.

A common property of the R1 elements in many *Drosophila* species, including *D. willistoni*, is that individual 28S genes contain multiple R1 insertions. The multiple R1s are organized in a tandem array at the target site with the individual copies separated by the 14 bp 28S gene target site duplication [22]. A search of the *D. willistoni* trace archive revealed that copies of R2/R1Dwi_SIDE were interspersed with the R1 elements in these tandem arrays. This result also strongly supports the conclusion that the hybrid SIDEs are mobilized like typical R1 elements.

PCR amplifications, similar to that in Figure 2C, were performed to estimate the relative abundance of the three hybrid SIDEs (data not shown). In *D. falleni*, R2/R1Dfa_SIDEs and R2 elements were present at approximately equal numbers; in *D. immigrans*, R2 elements outnumbered R2/R1Dim_SIDEs by a factor of 5; and in *D. innubila* only a few copies (less than 5) of the R2/R1Din_SIDE were detected. It should be noted that when multiple stocks from a species were sampled, R2 and R1 levels varied over a threefold to fivefold range [32,33]. Therefore, the SIDE levels detected in any one stock should not be regarded as characteristic for the species.

**Figure 6:**
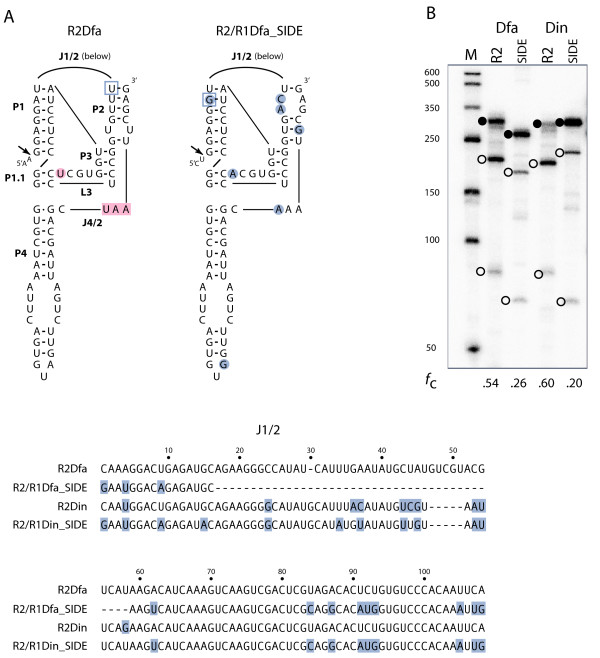
**The 5' ends of*Drosophila falleni*and*Drosophila innubila*elements function as ribozymes. (A)** The RNA secondary structures and highlighted nucleotides for R2Dfa and R2/R1Dfa_SIDE are as described in Figure 4. The corresponding regions in the *D. innubila* elements are identical except for the boxed U in R2Dfa (A in R2Din) and the boxed G in the R2/R1Dfa_SIDE (A in R2/R1Din_SIDE). J1/2 sequences for the elements are shown below with nucleotide differences relative to R2Dfa boxed in blue. **(B)***In vitro* cotranscription/cleavage assays as described in Figure 4.

The R2/R1 SIDEs presumably rely on an active ribozyme to process SIDE sequences from the R1 site within a 28S cotranscript. The ribozyme encoded in R2/R1Dwi_SIDE was shown capable of self-cleavage in Figure 4B. The secondary structures of and nucleotide differences between the 5' ends of the hybrid SIDE and R2 element from *D. falleni* are shown in Figure 6A. The single nucleotide differences between the elements found in *D. innubila* and *D. falleni* in the diagrammed regions are boxed. T7 *in vitro* transcription-cleavage assays revealed that the hybrid SIDEs from these two species showed self-cleavage levels between one-third and one-half the levels observed for the R2 elements (Figure 6B).

**Figure 7:**
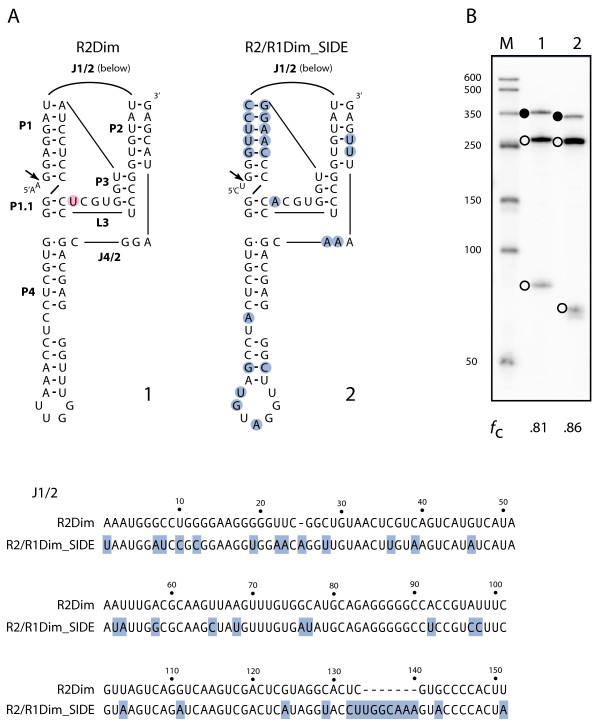
**The 5' ends of*Drosophila immigrans*elements function as ribozymes. (A)** Folded RNA secondary structures, J1/2 sequencers, and highlighted nucleotides for R2Dim and R2/R1Dim_SIDE are as described in Figure 4. **(B)***In vitro* cotranscription/cleavage assay as described in Figure 4.

Figure 7A shows a comparison between the 5' ends from the *D. immigrans* hybrid SIDE and R2 element. There are many nucleotide differences throughout the structure including a large number of compensatory changes in the P1 stem. The *in vitro* transcription-cleavage assays revealed that both the R2 and R2/R1 SIDE ribozymes self-cleaved at levels above 80% (Figure 7B). Therefore, the ribozymes encoded by the R2/R1 SIDEs in all four species can self-cleave and are likely able to process the 5' end of the element transcript out of the 28S cotranscript.

### *In vivo* template jumps

**Figure 8:**
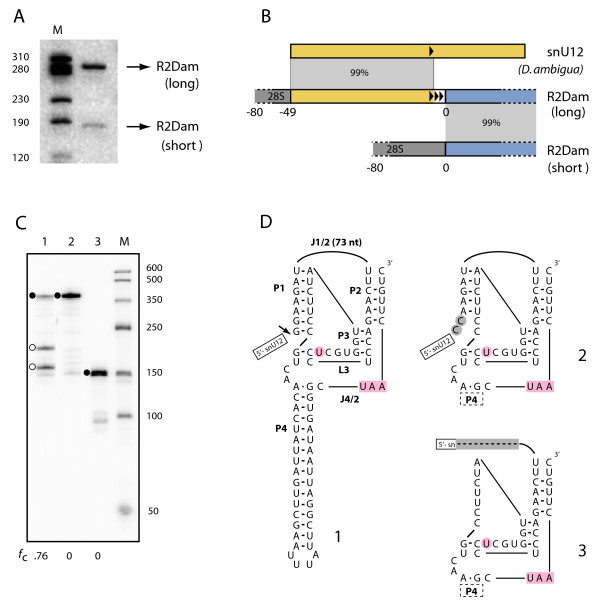
***In vivo*template jump to the small nuclear RNA, snU12. (A)** 5' R2 junction products from PCR amplification in *Drosophila ambigua* separated on a native, 8% polyacrylamide gel. Lane M, DNA length markers. **(B)** Diagrams of sequenced PCR products: 28S sequences (gray boxes); R2 sequences (blue boxes); snU12 sequences, yellow boxes. Long PCR products (12 clones) had a 48 bp deletion of upstream 28S sequences, 156 bp with sequence identity to the 5' end of snU12, and a 6 bp repeat at the snU12/R2 junction (arrowheads). Short products (eight clones) had typical 5' junctions that differed by zero to two non-templated nucleotides. **(C)***In vitro* cotranscription/cleavage assay of RNA containing R2 sequences with the snU12 extension indicated self-cleavage only immediately upstream of the R2 sequences (lane 1, open circles). RNA constructs (see (D)) designed to promote self-cleavage upstream of the snU12 sequences did not self-cleave (lanes 2 and 3, solid circles). **(D)** Secondary structures of R2 with U12 extension (1) and two modified constructs. The substitution of two C’s in the P1 stem (2) and deletion of the 5' end of R2 (3) are highlighted in gray. Structure number corresponds to lane number in (C). Nomenclature and highlighted nucleotides are as described in Figure 4.

During the attempts to identify SIDE families by PCR, R2 5' junction products that differed in length by 120 bp were observed in *Drosophila ambigua* (Figure 8A). The two junction types were confirmed using a second primer to sequences approximately 400 bp further downstream in the R2 element. Sequence analysis of cloned PCR products revealed the less abundant, shorter type to have typical R2 5' junctions (8 clones) while the more abundant, longer type contained a 48 bp deletion of the 28S gene and a 170 bp extension at the 5' end of R2 (12 clones). A sequence blast revealed this extension corresponded to the 5' end of the small nuclear RNA, snU12 [34]. Sequencing of the snU12 from *D. ambigua* revealed 99% identity to the first 156 bp of the R2 extension, and two additional copies of nucleotides 151 to 156 present in the R2 extension. The structures for the two junction types are diagrammed in Figure 8B.

The long variant of the R2 element likely originated during reverse transcription when the R2 reverse transcriptase jumped from the 5' end of the R2 RNA to snU12 RNA. This process has been described as a template jump and has been observed *in vitro* for the R2 reverse transcriptase [35] and *in vivo* for human L1 retrotransposition [36]. Unlike the reoccurring jumps to snU6 by L1 which gave rise to sequence variation [37,38], the multiple copies of R2 in *D. ambigua* are probably derived from a single jump to snU12 RNA since they all contain the same 6 bp repeats. Because this long form appears more abundant than the short form, one intriguing possibility is that a template jump gave rise to a new subfamily of R2 capable of retrotransposing with the upstream snU12 sequences.

If the 170 bp extension is retrotransposing with the R2 element, RNA self-cleavage should occur upstream of the U12 sequences rather than at the R2 5' junction. The products observed in T7 *in vitro* transcription-cleavage reactions are shown in Figure 8C. Efficient self-cleavage only occurred at the 5' end of the R2 sequences as observed for a typical *Drosophila* R2 ribozyme (Figure 8C, lane 1; Figure 8D, diagram 1). Two constructs were next generated in an attempt to force cleavage upstream of the U12 sequences. In the first, the two G’s at the base of the R2 P1 stem were mutated to C’s (Figure 8D, diagram 2); in the second, all but the first 12 bp of the snU12 sequence as well as the first 66 nucleotides at the 5' end of R2 were deleted (Figure 8D, diagram 3). Self-cleavage in standard *in vitro* reactions was not observed for either RNA construct (Figure 8C, lanes 2 and 3). We suggest the conditions needed for the self-cleavage of the R2 upstream of the snU12 extension are not met in our *in vitro* assay. We do not favor the alternative explanation that a single R2 insertion with U12 extension occurred in this species and was then duplicated multiple times by recombination. We have never seen high levels of amplification of a specific inserted rDNA unit in *Drosophila*.

Finally, two additional examples of template jumps were detected in *Drosophila* species. An 80 bp extension at the 5' junction of an R2 element was found in the trace archive of *D. pseudoobscura* (Additional file 2). These extra sequences differed at only one nucleotide position from the tRNA^lys(2)^ of this species. The presence of the nucleotides ‘CCA’ at the 3' end of this extension, which are added to tRNA post transcriptionally, confirm that the sequence arose by a jump from the R2 RNA template to the mature tRNA. Surveying the remaining 11 *Drosophila* trace archives for ‘CCA’ immediately upstream of full-length R2 insertions revealed another potential template jump to tRNA in *Drosophila yakuba*. In this case, 18 nucleotides from tRNA^gly^ were found at the 5' junction of an R2 (Additional file 2).

## Discussion

The experiments in this report provide evidence for new families of insertion elements in the 28S genes of *Drosophila*. Segments from R2 and/or R1 elements comprise these insertions, and they are mobilized by hijacking the R2 or R1 retrotransposition machinery. Because these non-autonomous elements rely (as does the R2 element itself) on cotranscription with the 28S gene, they are referred to as SIDEs rather than SINEs. Non-autonomous DNA-mediated transposable element families, such as the miniature inverted-repeat DINE-1 and non-autonomous P elements, have been previously documented in *Drosophila* genomes [39-41]. The R2 SIDE and R2/R1 hybrid SIDEs along with HeT-A [42] are, however, the only clear examples of non-autonomous retrotransposons to be found in *Drosophila*. Analysis of the SIDEs provides direct support for the model that R2 retrotransposition requires only the 5' end for RNA self-cleavage from a 28S cotranscript and the 3' UTR for binding the R2 protein to initiate TPRT. The discovery of SIDEs mobilized by the R1 machinery also provides strong support for the model [19] that the R1 protein recognizes the 3' UTR sequences/secondary structure of its RNA to initiate TPRT and thus belongs to the class of stringent non-LTR retrotransposable elements.

Because there is a single lineage of R2 element vertically transmitted in *Drosophila*[20], the levels of divergence between ribozyme sequences (excluding the highly variable J1/2 loop) from different elements can be compared to provide an estimate of the number of independently formed SIDEs and their approximate ages. First, the 25% sequence divergence between the ribozymes from the R2 element and R2 SIDE of *D. willistoni* is similar to the divergence between the ribozymes from the R2 elements from *D. willistoni* and *D. melanogaster* (23%) as well as between *D. ananassae* and *D. melanogaste*r (28%). Assuming similar levels of constraint on the ribozyme of these elements, this suggests the R2 SIDE lineage is as old as the divergence between species groups within the *Sophophora* subgenus, that is, over 40 million years [43,44]. Second, the 27% sequence divergence between the R2 and hybrid SIDE ribozymes from *D. immigrans* indicates the R2/R1Dim_SIDE lineage also dates back to a comparable time within the *Drosophila* subgenus. Third, the lower levels of sequence divergence between the ribozymes from R2/R1Dwi_SIDE and R2Dwi (11%) and between the ribozymes from R2/R1Dfa_SIDE and R2Dfa (10%) suggests both of these hybrid SIDEs have a more recent origin (approximately 20 million years ago). Because *D. falleni* and *D. willistoni* are in different subgenuses, their hybrid SIDEs arose independently. Finally, because R2/R1Dfa_SIDE and R2/R1Din_SIDE have only 3% sequence divergence, they likely represent the same event in the ancestor of these two closely related species. In summary, the five identified SIDEs in this report appear to have originated in four separate events.

Non-autonomous elements of DNA transposons (for example, miniature inverted-repeat transposable elements (MITEs)) and LTR retrotransposons (for example, terminal-repeat retrotransposons in miniature (TRIMs)) have been found to originate from autonomous elements by internal deletions [6,45-48]. The non-LTR, non-autonomous elements TbRIME and Ag-Sponge also appear to have arisen by internal deletions [49,50]. TbRIME is of special interest because it has sequence identity at the 5' end to the ribozyme encoded by L1Tc [31,51]. Two potential mechanisms could have formed the *Drosophila* SIDEs. First a template jump [35] during a retrotransposition reaction could have fused the 3' and 5' ends of an R2 element. The R2 5' junctions with upstream snU12 RNA and tRNA sequences shown in Figure 8 and Additional file 2 demonstrates the R2 protein does have the ability to template jump *in vivo*. In the case of the hybrid SIDEs, R1 sequences are located downstream of the R2 sequences, therefore, it is the R1 reverse transcriptase that must be postulated as responsible for the jumps. A second more likely possibility for the formation of the SIDEs is that non-homologous recombination within the rRNA gene locus joined the 5' end of R2 to either the 3' end of R2 or the 3' end of R1. Such recombinants could have been the result of DNA repair after retrotransposition events. The R2 machinery has been associated with large deletions of upstream rDNA sequences in *D. melanogaster*[52] and *D. simulans*[53]. Alternatively, the recombinations generating the SIDEs could simply have been aberrant versions of the frequent crossovers that give rise to the concerted evolution of the rDNA locus. Whatever the scenario, it seems unlikely that the SIDEs were formed in their present configuration. All SIDE families appear old, thus there has been ample opportunity for subsequent internal deletions to shorten the SIDEs until only the minimal sequences needed for activity remain.

Based on the sequence conservation of each SIDE, it appears that these elements have recently been active. Since their formation, the ribozymes and 3' ends of the SIDEs appear to be evolving similarly to the corresponding regions of R2 and R1 with two notable exceptions. A highly conserved ‘U’ located in the catalytic region of 18/19 *Drosophila* R2 ribozymes as well as in the R2 SIDE itself (pink circle, Figures 4A6A7A and 8D) has been substituted with an ‘A’ in all hybrid R1/R2 SIDEs. This substitution may reflect the difference in the insertion site of the hybrid SIDEs and consequently the upstream 28S sequences that must be cleaved from the cotranscripts. The second exception is a stop codon that is found in J4/2 in 18/19 R2 elements (pink box, Figures 4A6A and 8D) but not found in any of the five SIDEs. We suggest this stop codon is important in the initiation of translation of the R2 RNA open reading frame by way of an encoded internal ribosome entry site (IRES) [54,55], a function obviously not required for RNA arising from the SIDEs.

In general, non-LTR SIDEs appear to be rare. An L1 SIDE has not been observed despite the fact that studies of L1 retrotransposition in cultured cells revealed the generation of chimeric and internally deleted L1 insertions [38]. The cis preference of the L1 ORF2 protein for its RNA can, however, readily explain the absence of an associated SIDE [56]. Likewise, our survey of 39 *Drosophila* species suggests that the formation of R2/R1 hybrid SIDEs and to a greater extent R2 SIDEs is also rare and/or their survival after formation is unlikely. While there is no evidence that R1 and R2 undergo cis preference, our studies on R2 expression and regulation suggest an explanation for the paucity of R2 associated SIDEs [57,58]. Our current model suggests that *Drosophila* has the ability to select for transcription a localized region in the rDNA locus that has the lowest level of insertions. Because the SIDEs as well as the R2 elements rely on cotranscription with the 28S gene, their transcription can only occur whenever an rDNA unit with the insertion is located within this transcription domain. Consequently, in order for an R2 SIDE to retrotranspose both a copy of the SIDE and a copy of the autonomous R2 element must be present in the small transcription domain. Because the R2 lineage itself appears somewhat unstable and has been lost in several species of *Drosophila*[22,59], the survival of an R2 SIDE would be even more tenuous. However, R1 elements have been suggested to contain their own promoter and thus may not need to be within the transcription domain for activity. R1 elements are present in all lineages of *Drosophila* and indeed many species have two distinct lineages [21,59]. The greater evolutionary stability of the R1 retrotransposition machinery and the independence of transcriptional control of the hybrid SIDE from the autonomous R1 elements may explain why these SIDEs appear to have a greater chance of long-term survival within the locus.

## Conclusion

This report demonstrates that R1 and R2 elements, like many other non-LTR retrotransposons, are parasitized by non-autonomous sequences that hijack their retrotransposition machinery. These short internally deleted elements, or SIDEs as we have called them, need only the R2 self-cleaving ribozyme at their 5’ end to process themselves from a 28S rRNA co-transcript and 3’ RNA sequences which can be bound by the retrotransposition machinery of an autonomous element. These R2 SIDEs and R2/R1 SIDEs can survive only as long as the autonomous R1 and R2 elements are able to survive. The existence of each element would seem tenuous, as there are a limited number of potential insertion sites in the rDNA locus. However, the high rates of recombination and turnover of rDNA units within this locus facilitates mobile element survival [20,21,57,59]. The finding that some lineages of the SIDEs have persisted for an estimated 40 million years suggests this genomic niche is sometimes even flexible enough to maintain the parasites of R1 and R2.

## Methods

### PCR amplification/cloning/nucleotide sequence determination

Genomic DNA from most *Drosophila* species surveyed was previously isolated [20,21]. For *D. innubila* and *Drosophila phalerata*, genomic DNA was isolated from adult flies (a gift from J Jaenike) as described in the above references. The initial survey for R2 SIDEs was performed using two primers to the conserved catalytic region of the R2 ribozyme (R2(catA), 5'-AAAACCTCCTCGTGGTRTY-3') and (R2(catB), 5'-GTGGCCTCCTCGTGGTRTY-3') separately paired with a reverse primer which anneals to the 28S gene 29 to 50 nucleotides downstream of the R2 site (28S(+50), 5'-CGTTAATCCATTCATGCGCGTC-3'). The survey for R2/R1 hybrid SIDEs was performed using a reverse primer to the conserved catalytic region of the R2 ribozyme (R2 (cat1), 5'-RAYACCACGAGGAGG-3') paired with a primer annealed to the 28S gene 1 to 15 nucleotides downstream of the R2 insertion site (28S (+15), 5'-TAGCCAAATGCCTCG-3'). A second survey for R2 SIDEs and R2 5' variation was performed by pairing the R2 (cat1) primer with a 28S gene primer 81 to 61 nucleotides upstream of the R2 insertion site (28S (−81), 5'-TGCCCAGTCCTCTGAATGTC-3'). Where noted R corresponds to A and G; Y corresponds to C and G; and W corresponds to A and T. PCR fragments were cloned into the pCR2.1-TOPO cloning vector (Invitrogen, Grand Island, NY USA) and sequenced (Macrogen, Rockville, MD USA).

The 3' ends of the R2/R1 SIDEs were obtained by pairing the *D. falleni/D. innubila* primer (fal(J1/2), 5'-GCACATGGTGTCCCACAAATTGTCAG-3') and the *D. immigrans* primer (imm(J1/2), 5'-TACCTTGGCAAAGTACCC-3') with a reverse primer which annealed to the 28S gene 6 to 27 nucleotides downstream of the R1 site (28S(+80), 5'-GTTCCCTTGGCTGTGGTTTCGC-3'). The 3' end of the R2 ribozyme from *D. ambigua* was obtained by pairing primer (Cys(amb), 5'-CATRTGNACRCCNARNCC) with (28S(−81)). A partial snU12 sequence from *D. ambigua* was obtained by pairing primers: (DpsU12up, 5'-GTGCCTGAAATTAATGAGTAAGG) and (DpsU12down, GGGCAGATCGCAAACACCC). All PCR products were cloned and sequenced as above.

The primers to sequences shared by the R2 element and SIDE(s) in *D. willistoni* (Cons(wil), 5'-ACACCACGAGGAGGTTTCG-3'), in *D. falleni/D. innubila* (Cons(fal), 5'-ACACTGAATTTAGCACCCGGAGG-3'), and in *D. immigrans* (Cons(imm), 5'-ACGGWGGCCCCCTCTGC-3') were paired with either 28S(−81) or (28S(−32), 5'-CAACGGCGGGAGTAACTATG-3') to determine relative SIDE abundance. PCR products were separated on 8.75% polyacrylamide gels and ethidium bromide stained bands analyzed using QuantityOne (BioRad, Hercules, CA USA).

### Template generation

Reverse primers which annealed to sequences downstream of the SIDE ribozymes: *D. willistoni* (R2SIDE(wil), 5'-AGGATTAGACCTTCAGAATACC-3') and (R2/R1SIDE(wil), 5'- GCCAAACAGGAAATGGGTAAACC-3') *D. falleni/D. innubila* (R2/R1SIDE(fal), 5'-CTACCAATTCTAACTCCAAAACAG-3'), and *D. immigrans* (R2/R1SIDE(imm), 5'-TATGGAAGAATTCTAACCCGC-3') as well as downstream of the R2 elements: *D. willistoni* (R2(wil), 5'-GGTAACCCCAAGAGTTGCTTC-3'), *D. falleni/D. innubila* (R2(fal), 5'-TTGGGTAGGTAACCCTTTGGAC-3'), *D. immigrans* (R2(imm), 5'-TGATTTGCACCAACAGTTGTC-3') and *D. ambigua* (R2(amb), 5'-CCCCATAGGACTGTTTCGCTG-3') were paired with a 28S upstream primer containing a T7 promoter (28S(−95), 5'-TAATACGACTCACTATAGGGCACAATGTGATTTCTGCCCAGT-3'). PCR fragments were cloned into the TOPO cloning vector (Invitrogen, Grand Island, NY USA). DNA templates were generated by PCR amplification using the same primers with unincorporated primers and nucleotides removed with the PCR Purification Kit (BioBasics, Markham, Ontario Canada).

### Cotranscription/cleavage assay

Assays were preformed as described in [25]. Approximately 0.1 μg of PCR template was incubated in transcription buffer with 20 units of T7 RNA polymerase (Fermentas, Glen Burnie, MD USA) and trace amounts of [α-^32^P]UTP) for 1 h at 42°C. Reactions were then placed on ice and four volumes of 95% formamide, 10 mM EDTA (pH 8) added. RNA products were denatured at 92°C for 3 minutes and separated on 8 M urea, 5% polyacrylamide gels. The dried gels were exposed to a phosphorimager screen and analyzed using QuantityOne (BioRad, Hercules, CA USA).

### SIDE sequence files

Complete nucleotide sequences for each SIDE can be found in Additional file 3 (R2Dwi_SIDE), Additional file 4 (R2/R1Dwi_SIDE), Additional file 5 (R2/R1Dfa_SIDE), Additional file 6 (R2/R1Din_SIDE), and Additional file 7 (R2/R1Dim_SIDE). Sequences were aligned with the aid of ClustalX [60].

### Original sequence reads

Sequencing reads from the whole genome shotgun sequencing project of *D. willistoni* (8.4-fold coverage), *D. pseudoobscura* (ninefold coverage), and *D. yakuba* (ninefold coverage) were accessed by Blast search (version 2.2.17) in the trace archives at NCBI [26].

## Competing interests

The authors declare that they have no competing interests.

## Authors’ contributions

DGE carried out the studies and drafted the manuscript. THE participated in the design of the studies and helped finalize the manuscript. Both authors read and approved the final manuscript.

## Supplementary Material

Additional file 1 **R1 and hybrid SIDE 3' end sequence conservation.** Two lineages of R1 elements, R1A and R1B, suggested to have diverged over 100 million years ago and maintained in *Drosophila* by vertical descent were previously found to have little sequence conservation in the 3' untranslated regions (UTRs). Shown in this figure are sequences from the 3' ends of nine R1A and six R1B family members that represent the diversity of *Drosophila*. The six R1 segments with the highest levels of identity were also identifiable in the four families of R2/R1 SIDEs. Distances from the stop codon of open reading frame 2 (ORF2) (R1 elements) or the ribozyme (SIDE elements) as well as distances between conserved segments are shown in parentheses. Dmer, *Drosophila mercatorum*; Dfa, *Drosophila falleni*; Dte, *Drosophila testacea*; Dpu, *Drosophila putrida*; Dan, *Drosophila ananassae*; Dta, *Drosophila takahashii*; Dme, *Drosophila melanogaster*; Dps, *Drosophila pseudoobscura*; Dvi, *Drosophila virillis*; Dre, *Drosophila recens*; Dgr, *Drosophila grimshawii*. Click here for file

Additional file 2 **Template jumps to tRNA. (A)** Diagram of an R2 5' junction found in the *Drosophila pseudoobscura* trace archive indicating a template jump from R2 RNA to tRNA^lys(2)^: R2 (blue box); tRNA (purple box); 28S gene (gray box). Partial 28S and R2 junction sequences and the entire tRNA^lys(2)^ sequence is shown below the diagram. Three non-templated nucleotides (white box) are present between the tRNA and 28S sequences. **(B)** Diagram and sequence of the 5' junction of a template jump to tRNA^gly^ found in the *Drosophila yakuba* trace archive. Shading as in **(A)**. Click here for file

Additional file 3 R2Dwi_SIDE sequence. Click here for file

Additional file 4 R2/R1Dwi_SIDE sequence. Click here for file

Additional file 5 R2/R1Dfa_SIDE sequence. Click here for file

Additional file 6 R2/R1Din_SIDE sequence. Click here for file

Additional file 7 R2/R1Dim_SIDE sequence. Click here for file
