# Supplementary material for: R2 and R2/R1 hybrid non-autonomous retrotransposons derived by internal deletions of full-length elements
Source: Mob DNA. 2012 May 23;3:10. doi: 10.1186/1759-8753-3-10 (PMC3414825; doi:10.1186/1759-8753-3-10)
Supplement: Additional file 1 — R1 and hybrid SIDE 3' end sequence conservation. Two lineages of R1 elements, R1A and R1B, suggested to have diverged over 100 million years ago and maintained in Drosophila by vertical descent were previously found to have little sequence conservation in the 3' untranslated regions (UTRs). Shown in this figure are sequences from the 3' ends of nine R1A and six R1B family members that represent the diversity of Drosophila. The six R1 segments with the highest levels of identity were also identifiable in the four families of R2/R1 SIDEs. Distances from the stop codon of open reading frame 2 (ORF2) (R1 elements) or the ribozyme (SIDE elements) as well as distances between conserved segments are shown in parentheses. Dmer, Drosophila mercatorum; Dfa, Drosophila falleni; Dte, Drosophila testacea; Dpu, Drosophila putrida; Dan, Drosophila ananassae; Dta, Drosophila takahashii; Dme, Drosophila melanogaster; Dps, Drosophila pseudoobscura; Dvi, Drosophila virillis; Dre, Drosophila recens; Dgr, Drosophila grimshawii. [file 1759-8753-3-10-S1.pdf]

```
                          GGTACC-ACGGGT(xx bp)GGGAGTATCGTGGTGGTTG-TGGTT(xx bp)GAGTTGCGTT( xx bp)AGAAGAGGTGTTAGATAGGCCT-CGCTCCTCACCAAGG
R1DmerA       (646 bp)T.A...-.....A(44 bp)......T............-....-(25 bp).C....AA.C(  5 bp)..-C.GA.GT.A..........-...C...G......A
R1DfaA        (401 bp).....-.......(45 bp)......-............-.....( 5 bp).C........( 10 bp)...GA..T..C.....GC--T.-...CT.A........
R1DteA        (295 bp)..A...C......(44 bp)A.....-............-..TA.( 6 bp).C........( 10 bp)...GA.AC..C.....GT--T.-..GC...........
R1DpuA        (534 bp)..A...-......(44 bp)..-...-......G.....-....-( 6 bp).C........( 11 bp)...GAG.C..C.....GC-TTC-GA.C.T..GT.....
R1DanA        (142 bp)......-......(43 bp)...................-.....(39 bp)..........( 30 bp)..T.......A......CA.T.-...............
R1DwiA        (704 bp).....T-.....A(47 bp).....-.....A....C..-.....(39 bp)..........( 28 bp)..C......T......G.....-.......T...C...
R1DtaA        (144 bp)......-......(43 bp)...................-.....(39 bp).........C( 30 BP)..T..G.A.T.-......AT.C-T........G.....
R1DmeA        (145 bp)......-......(43 bp)...................-.....(40 bp).....A..G.( 39 bp)..T....A.T....G...AT..-.........G.....
R1DpsA        ( 30 bp)......-......(41 bp)...................-.....(43 bp)........C.( 28 bp)G.....C..........CA...-...C...........

R1DviB        (171 bp)......-......(53 bp)...................-.....(36 bp).........C( 29 bp)G.......C-........T...-..TG..AA.......
R1DreB        (537 bp)......-......(51 bp)...................-.....(35 bp)..........( 30 bp)G.C.....A-....G.......-...............
R1DteB        (603 bp)...G..-......(53 bp)...................-.....(37 bp)..CC......( 30 bp)..T......T....G.......-..A............
R1DgrB        (482 bp)......-......(55 bp)......T............-.....(38 bp)TT......C.( 29 bp)G....TC..T....G.....GG-.C....AA.......
R1DanB        (537 bp)......-......(50 bp).....CTA...........-.....(38 bp)..........( 30 bp)G........-............C.AT..T.A...G...
R1DimB        (239 bp)......-.....A(51 bp)................C..-.....(37 bp)..........( 30 bp)..C......-A...........-.......T......

R2/R1Dwi_SIDE ( 85 bp).....T-.....A(43 bp).....-.....A....C..-.....(37 bp).......AAA( 25 bp)..C......T....G.C.....-...CT...-.....A
R2/R1Dfa_SIDE ( 88 bp)......-......(52 bp)................C..A.....(39 bp).........A( 30 bp)G......A.T.A.......T..-..GC...........
R2/R1Din_SIDE ( 88 bp)......-......(52 bp)..C................A.....(39 bp).........A( 30 bp)GA.....A.T.A.......T..-..GC...........
R2/R1Dim_SIDE (122 bp)...-..-.....G(42 bp)T...C--............-.....(34 bp)T....TG...(284 bp)T........-.GC.C.G.....-.......T....G.A


              ( xx bp)TACCGTGGTTGT-AATCC(xx bp)GGAACACGCC-ACGTTAAACA(xx bp)
R1DmerA       (121 bp)............-..A..( 8 bp)..T.......-..........(13 bp)
R1DfaA        (129 bp).........C..-.....( 5 bp)..........-..........(22 bp)
R1DteA        (126 bp)............-.....( 6 bp)....G.....-........T.(23 bp)
R1DpuA        (127 bp)............-.....( 6 bp)A...G.....-........T.(22 bp)
R1DanA        (142 bp).G..........-.....( 9 bp)..........-..........(27 bp)
R1DwiA        (135 bp)............-..A..( 9 bp)..T.....T.-........T.(12 bp)
R1DtaA        (135 bp).G..........-.....(10 bp)......T...-........T.( 9 bp)
R1DmeA        (151 bp).G..........-.....( 9 bp)..........-....A...T.(28 bp)
R1DpsA        (139 bp)CG.........A-.....( 8 bp).....-....-....A...T.(23 bp)

R1DviB        ( 95 bp)A......AGAT.-..G..( 5 bp)..C.GGT..TC.......G.C(12 bp)
R1DreB        ( 94 bp)C......AGAT.-..G..( 5 bp)..C.GGA..TC..........(12 bp)
R1DteB        ( 91 bp)C......AAAT.-..G..( 5 bp)..C.GGA.TTC..........(12 bp)
R1DgrB        (101 bp)C......AGAT.-..G..( 5 bp)..C.GGT..TC.........C(12 bp)
R1DanB        (109 bp)GG.....AGAT.-..G..( 5 bp)..C.GGT..TC..........(13 bp)
R1DimB        (102 bp)C......AGATC-..G..( 5 bp)..C.GGT.TTC....A....C(14 bp)

R2/R1Dwi_SIDE (134 bp).G.......A..T..A..( 9 bp)..T.......-....A.....(12 bp)
R2/R1Dfa_SIDE (118 bp).........A..-.....( 6 bp)..C.......-........T.(23 bp)
R2/R1Din_SIDE ( 93 bp).........A..-.....( 6 bp)..C.......-........T.(23 bp)
R2/R1Dim_SIDE (100 bp)C......AGATC-..G..( 5 bp)..C.GGT.TTC....A....C(13 bp)
```

Additional File 1
