# Supplementary figures and images for: R2 and R2/R1 hybrid non-autonomous retrotransposons derived by internal deletions of full-length elements
Source: Mob DNA. 2012 May 23;3:10. doi: 10.1186/1759-8753-3-10 (PMC3414825; doi:10.1186/1759-8753-3-10)

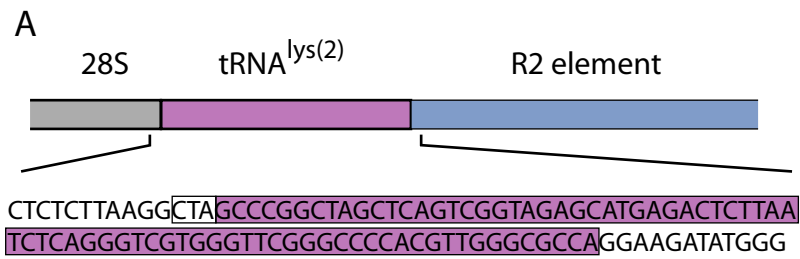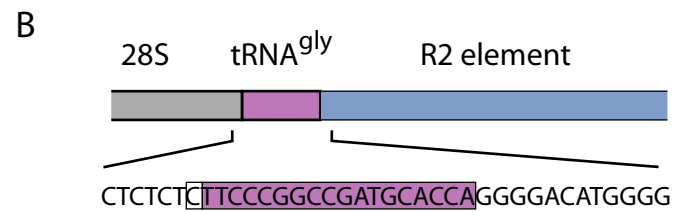

Supplement: Additional file 2 — Template jumps to tRNA. (A) Diagram of an R2 5' junction found in the Drosophila pseudoobscura trace archive indicating a template jump from R2 RNA to tRNAlys(2): R2 (blue box); tRNA (purple box); 28S gene (gray box). Partial 28S and R2 junction sequences and the entire tRNAlys(2) sequence is shown below the diagram. Three non-templated nucleotides (white box) are present between the tRNA and 28S sequences. (B) Diagram and sequence of the 5' junction of a template jump to tRNAgly found in the Drosophila yakuba trace archive. Shading as in (A). [file 1759-8753-3-10-S2.pdf]
